# A Survey of Path Loss Prediction and Channel Models for Unmanned Aerial Systems for System-Level Simulations

**DOI:** 10.3390/s23104775

**Published:** 2023-05-15

**Authors:** Nektarios Moraitis, Konstantinos Psychogios, Athanasios D. Panagopoulos

**Affiliations:** School of Electrical and Computer Engineering, National Technical University of Athens, 9 Heroon Polytechniou Str., Zografou, 15773 Athens, Greece; konpsychogios@gmail.com (K.P.); thpanag@ece.ntua.gr (A.D.P.)

**Keywords:** air-to-air (A2A), air-to-space (A2S), path-loss prediction, channel modeling, propagation study, unmanned aerial systems (UAS)

## Abstract

Unmanned aerial systems (UAS) have recently gained popularity, and they are envisioned as an integral parts of the current and future wireless and mobile-radio networks. Despite the exhaustive research on air-to-ground channels, there are insufficient studies, experimental campaigns and general channel models related to air-to-space (A2S) and air-to-air (A2A) wireless links. This paper presents a comprehensive review of the available channel models and path-loss prediction for A2S and A2A communications. Specific case studies attempting to extend current models’ parameters and provide important knowledge of the channel behavior in combination with UAV flight characteristics are also provided. A time-series rain-attenuation synthesizer is also presented that describes quite accurately the impact of the troposphere at frequencies above 10 GHz. This specific model can be also applied to both A2S and A2A wireless links. Finally, scientific challenges and gaps that can be used for future research on the upcoming 6G networks are highlighted.

## 1. Introduction

On a global scale, there has been an unprecedented deployment of the unmanned aerial system (UAS) for diverse applications, both civil and military [1]. The UAS is a general term that incorporates unmanned aerial vehicles (UAVs), informally known as “drones”, as well as ground-control stations and communication links [2]. There are many UAV categories, distinguished by their endurance, range, and size, parameters that determine their operational use. Furthermore, UAVs are considered technological enablers, fulfilling the challenging requirements of the new services provided by future sixth-generation (6G) networks. More specifically, aerial platforms are regarded as integral parts of a ubiquitous communication scenario, in which they are foreseen to provide three-dimensional (3D) cellular coverage in 6G networks. The main concept is to operate an abundance of these platforms, comprising UAVs, high-altitude platforms (HAPs), and constellations of very-low-earth-orbit (VLEO) satellites, so as to deliver cloud services with tolerable delays [3].

**Figure 1:**
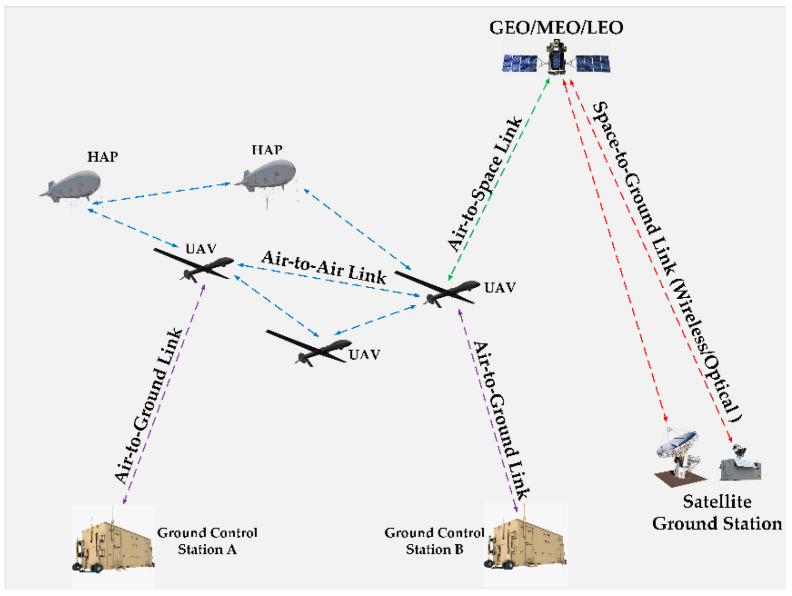
UAS-connectivity scenarios.

In this context, potential connectivity scenarios for UAV applications are presented in Figure 1, in which three types of communication link are identified: (i) air-to-ground (A2G), (ii) air-to-air (A2A), and (iii) air-to-space (A2S) channels (the space-to-ground link is beyond the scope of this article). These three cases are exploited for the communication between UAVs and ground-control stations (for guidance, telemetry, and data transfer), for communication between many UAVs (for swarm applications or data relay), and, finally, for the communication between UAVs and GEO, MEO or LEO satellites (for navigation purposes or data relay). The A2S channel is also exploited for beyond-line-of-sight (BLOS) control and non-payload communication (CNPC) links. Non-payload links are used for command and control, whereas the payload is the data transferred from cameras (e.g., the real-time video feed from a thermographic camera) or other measuring equipment [4]. These established communication links are examined as parts of flying ad hoc networks (FANETs), which envisage the networking and cooperation of multiple UAVs [5]. The introduced channels are the key elements in the design of proper UAV datalinks and protocols for heterogeneous networks and system-level simulations.

The frequencies allocated for the aforementioned communication links, including CNPC and payload links, are in the license-free spectrum at the segments of 2400–2483.5 MHz, 5470–5600 MHz, 5650–5725 MHz, and 5725–5875 MHz, in which specific restrictions apply that concern the effective isotropic radiated power (eirp). Furthermore, specific regulations apply in which, according to the International Telecommunications Union (ITU) and the International Civil Aviation Organization (ICAO), a specific protected spectrum is allocated in the L-band (960–977 MHz) and the C-band (5030–5091 MHz) for the CNPC link [6]. Furthermore, the Ku-band-frequency segments of 10.95–12.75 GHz (downlink), and 14.0–14.47 GHz (uplink), as well as the Ka-band segments of 19.70–20.20 GHz (downlink), and 29.5–30 GHz (uplink), are also authorized for BLOS and CNPC links of satellite aeronautical communications [5]. In addition, ITU has specific spectrum segments allocated at 13.25–13.40 GHz, 15.4–15.7 GHz, 22.5–22.55 GHz, and 23.55–23.60 GHz, for frequency sharing between UAS, for BLOS and CNPC communication links, as well as other existing or planned services in these bands [7]. Finally, the total required bandwidth for operational line-of-sight (LOS) terrestrial links is 34 MHz, whereas for BLOS satellite systems, a bandwidth of 56 MHz is recommended [4].

In this context, to ensure robustness and reliability for the operation of UAVs, it is essential that appropriate and versatile channel-propagation models are studied and developed for each connectivity scenario. The cornerstone of successful rudimentary network planning and establishment is to estimate accurately the propagation loss of a wireless link, thus ensuring robust, secure, and convenient communication between the terminals [8]. Path loss is an important large-scale channel characteristic that, together with shadow fading, defines the variation in signal strength over the Tx–Rx distance. Therefore, it is essential that accurate models are employed and evaluated to provide theoretical guidance and assist not only in coverage estimation, but also in frequency allocation and interference assessment [9]. Path-loss models are simpler and more easily applicable than geometrical stochastic models, for example. This study focuses not only on providing existing path-loss models, but also on their use in different scenarios and, in many cases, the extension of their applicability. Consequently, an accurate path-loss estimation should be inserted in the link-budget calculations of unmanned aerial vehicles in system-level simulations. Finally, the application of rigorous path-loss models is crucial, since path-loss miscalculations may compromise the study of the wireless link, affect the service-range estimation, lead to outage, and extract misleading interference results.

In the frame of the A2G channel, there has been extensive research in the past years, in which various channel propagation models were developed based on measurement campaigns, simulations, studies, and surveys ([1,2,10,11], and the references therein). In contrast to previous research, this article highlights all the recent trends in propagation-channel modeling for UAVs, emphasizing state-of-the art research explicitly for the A2S-and-A2A communication channel. The additional contributions of this survey are as follows:

- A comprehensive survey is delivered in which available state-of-the-art channel-propagation models are studied. The presented models are retrieved from available experimental campaigns or from theoretical studies and simulations regarding the A2S-and-A2A wireless channel.
- Specific cases studies are provided that deliver system-level simulations in order to evaluate and assess the performance of selected path-loss models based on their fundamental parameters. Further, an attempt is undertaken to extend current models’ attributes and gain valuable information on the channel behavior in conjunction with the UAS flight characteristics.
- A modified time-series-attenuation synthesizer is introduced, which evaluates the induced excess loss of precipitation in the Ku and Ka bands. Valuable simulated results are provided in terms of rain attenuation and exceedance probability. The specific stochastic model can be applied in both A2S and A2A links.
- This article suggests future research directions and areas of interest and describes the potential challenges in the modeling of the UAS-propagation channel that stem from the inherent nature of the wireless link, or from the structure of the UAV itself. Finally, antenna diversity and interference-related issues are also introduced, along with potential new measurement campaigns.

The methodology of this contribution and the performance of the survey were based on a systematic literature review using journals’ and conference proceedings’ databases (e.g., IEEE, Elsevier, MDPI, etc.) with specific keywords related to path-loss prediction, air-to-air, and air-to-space channel models for unmanned aerial systems. When screening and filtering the outcomes of the literature search, air-to-ground models were excluded and are not presented in this study, since there are many surveys on this specific subject. The search was conducted from 1973 to 2023 to ensure a comprehensive review of the literature in this area, with a greater emphasis placed on the most recent studies. Another aim was to present models that can be employed for system-level simulations of UAS in various geometries and scenarios. We focused on A2S and A2A channel models in experimental campaigns, theoretical studies and simulations, as well as rain-attenuation time-series synthesizers.

**Figure 2:**
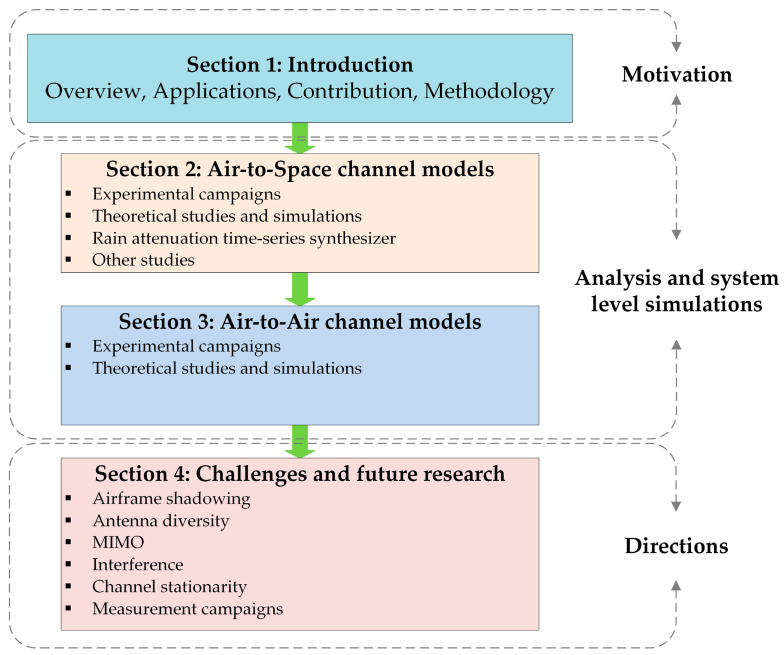
Article layout and organization.

Figure 2 illustrates the article’s layout and a brief summary of the organization of its main content. More specifically, Section 1 provides an overview and the applications of the UAS, as well as the main motivation of this survey. The available experimental campaigns and theoretical studies regarding the A2S communication link are described analytically in Section 2. Additionally, a rain-attenuation time-series synthesizer that can be applied in both the A2S and the A2A link is presented. State-of-the art channel models for the A2A wireless link are introduced in Section 3, based either on measurement campaigns or on theoretical studies. System-level simulations were conducted, producing interesting results and conclusions regarding the channel behavior. Section 4 highlights the potential challenges and addresses future research directions in the fields of airframe shadowing, interference, antenna diversity, channel stationarity, and measurement campaigns. Finally, a brief summary of this work is given in Section 5.

## 2. Air-to-Space Channel Models

The interconnection of UAV communication networks with traditional satellite and terrestrial cellular networks constitutes the space–air–ground integrated network (SAGIN) [12], a heterogeneous network that supports seamless coverage and enhanced capacity, making 3D architecture a reality for established 6G networks. Therefore, to realize these networks, it is of utmost importance to have a thorough knowledge of the A2S propagation channel. There are no potential scatterers close to the terminals of the A2S link, yet the atmospheric effects, the influence of the UAV structure, the elevation angle to the satellite, or even the scattering that originates from the ground may cause the harsh fading of the received signal, which implies the need for the rigorous study and modeling of the A2S channel.

### 2.1. Experimental Campaigns

The A2S link was investigated in [13], in terms of airframe shadowing events. More specifically, experiments took place with a manned aircraft receiving the pilot-tone signal from a satellite (Italsat beacon) at a frequency of 18.685 GHz. The results revealed that in the LOS condition, no shadowing was induced during the flight, and the received signal was modeled by the Rician probability density function (pdf) with the K-factor reaching almost 34 dB. By contrast, in non-LOS (NLOS) cases, the received signal exhibited both multipath fading and shadowing effects, in which the signal level depended directly on the flight maneuvers. For example, when the aircraft formed a 25-degree roll angle when performing a typical U-turn, the signal level decreased, since the wing compromised the received signal. Furthermore, in extreme scenarios, in which 45-degree rolls were tested, signal-shadowing events up to 12.5 dB occurred when the wing crossed the LOS level. Another observation was the difference of 0.78 dB in the signal reception when the aircraft flew above and below the clouds. Finally, when the aircraft flew in a wavy line, in line with the propagation path from the satellite, there was a periodical block of the propagation path from the satellite from the aircraft’s tail. This accounted for the diffraction and shadowing incidences with fade depths of up to 2–3 dB.

Throughput- and latency-measurement tests between a satellite and a UAV were carried out in [14], in order to assess the reliability and robustness of the A2S link and investigate the potential of a satellite network to remotely control a BLOS drone. For this purpose, the Inmarsat I4 satellite network was exploited, along with an Aviator 200 UAV moving at a speed of 140 km/h. To test the throughput, the download time of a 1.2-MB file was examined using a continuously transmitting channel with a data rate of 200 kbps. The measurements took place in the greater area of Clinceni in Romania, and data were collected at 24 preselected waypoints. The test outcome revealed that the file downloading was completed with an average throughput of 140.4 kbps and an average latency time of 254.2 ms.

**Figure 3:**
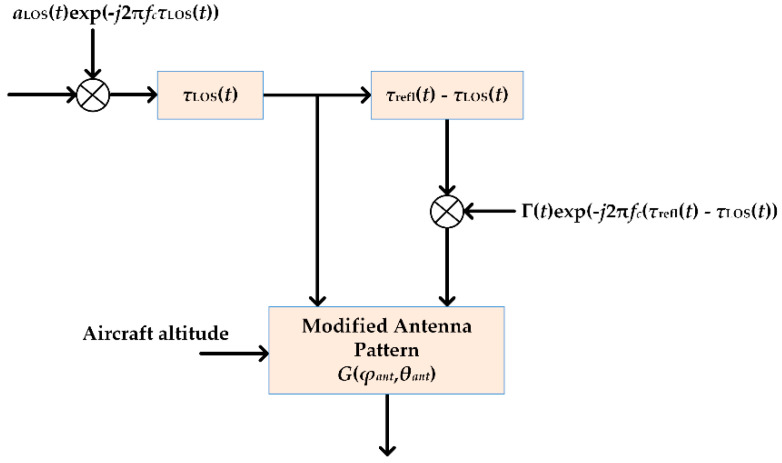
Model layout of the A2S channel [15].

Measurements of the A2S channel at the L-band (1575.42 MHz) using four different aircraft types were conducted in [15]. A dual polarized antenna array (i.e., an array with right- and left-hand circular polarized hemispherical patch antennas) was mounted on top of the aircraft to receive signals from the satellite, and a 2 × 2 antenna array was applied on the bottom of the aircraft in order to capture the multipath signals reflected from the ground. The addressed channel model was based on a physical–statistical approximation, which took into account, in addition to the LOS component between the satellite and the aircrafts, the ground-reflected component, estimating for this purpose the ground-reflection coefficient. The specific parameter was determined as a function of the elevation angle and its statistical parameters (mean and standard deviation) for both polarizations, which are available in [15]. The corresponding layout of the final channel model is provided in Figure 3.

### 2.2. Theoretical Studies and Simulations for Channel Modeling

The first theoretical study regarding the aeronautical channel of an A2S link was conducted in 1973 [16], emphasizing the effects of the indirect paths reflected and scattered by the Earth surface. The channel was modeled by employing three distinct coefficients. These incorporated the LOS path, which is the direct path between two aircraft, the specular reflection component, which is the shortest path between the two aircraft through the underlying terrain, and the scattered paths, which included all the scattered components from the ground apart from the specular reflection.

A channel model that predicts path loss for an A2S link, studying the propagation characteristics in the THz band, was proposed in [17]. In addition to the terrestrial geometrical characteristics, the suggested model considered the atmospheric characteristics of the THz propagation channel, such as rain, clouds, and non-homogeneous molecular-absorption losses. The combined path-loss model is provided by

(1) $$PL(f,r)=\frac{{\left(4\pi r_{as}f\right)}^{2}\delta_{\mathrm{rain}}(r_{r})\delta_{\mathrm{cloud}}(r_{c})}{c^{2}G_{\mathrm{Tx}}(\theta_{\mathrm{Tx}})G_{\mathrm{Rx}}(\theta_{\mathrm{Rx}})}e^{\int_{r_{1}}^{r_{2}}\kappa_{\alpha }(f,r_{\mathrm{atm}})dr_{\mathrm{atm}}},$$

where *G*_Tx_(*θ*_Tx_) and *G*_Rx_(*θ*_Rx_), are the transmitter (Tx) and receiver (Rx) antenna gains at the pointing angles *θ*_Tx_ and *θ*_Rx_, respectively, *r_as_* is the direct distance between the UAV and the satellite, *f* is the operating frequency, and *c* is the speed of light. Furthermore, *δ*_rain_(*r_r_*) and *δ*_cloud_(*r_c_*) are the rain and cloud losses, where *r_r_* and *r_c_* are the distance of the path within the rain and clouds, respectively. The rain loss is calculated according to the ITU-R P.838-3 recommendation, on the basis of the following expression:

(2) $$\delta_{\mathrm{rain}}(r_{r})=kR_{r}^{a}r_{eff},$$

where *k* and *α* can be resolved according to [18], *R_r_* is the rain rate in mm/h, and *r_eff_* is the effective distance. The cloud loss can be determined from the ITU-R P.840-8 model, according to

(3) $$\delta_{\mathrm{cloud}}(r_{c})=K_{l}Mr_{c},$$

where *M* is the liquid-water density in the cloud or fog in g/m^3^, *K_l_* is the cloud-liquid-water specific attenuation coefficient in (dB/km)/(g/m^3^), and *r_c_* is the distance of the path through clouds/fog [19]. Finally, the *k_a_*(*f*,*r*_atm_) in (1) is the molecular absorption coefficient for non-homogeneous atmospheric conditions. The specific parameter depends on the pressure, the temperature, and the molecular composition of the propagation path through the atmosphere. Its calculation is not straightforward, and can be resolved from molecular absorption databases, as in [17] and references therein. For the simulation process, the airplane- and satellite-antenna diameters were set as 0.5 and 1 m, respectively, with an aperture efficiency of 70%. These resulted in a combined (airplane plus satellite) gain between 191 and 150 dBi, depending on the frequency. Two different satellite orbits were tested, a GEO (35,786 km) and a LEO (500 km), which both corresponded to elevation angles in the range of 12°–90° (from the horizon of the airplane to the satellite). The excess loss due to rain and clouds reached up to 25 dB (at about 1000 GHz). Furthermore, for an airplane flying at 11 km, the path loss was severe for both the LEO and the GEO orbit, varying, on average, between 190 and 260 dB (for frequencies between 0.1 and 3 THz), presenting peaks at specific frequency instants due to the molecular absorption. These path-loss peaks reached up to 300 dB. However, it is worth noting that these simulation tests did not take into account the excess loss due to rain or clouds. These observed path-loss values indicate the necessity of using large-aperture antennas, as in those previously mentioned, at both terminals, in order to compensate for the considerable loss and establish successful links. Another observation was the path-loss variation as a function of the airplane altitude. The simulation results revealed that at high-altitude links, the free-space loss dominated the signal propagation. However, the excess loss from the molecular absorption increased with frequency, even at lower altitudes, implying that A2S links demonstrate better performance at higher airplane heights. Based on the results, the THz band is promising for A2S and S2A links, although these should be limited at frequencies below 380 GHz in order to effectively compensate path loss, provided that antennas with sufficient apertures are employed. Finally, it should be mentioned that additional losses may be encountered due to weather phenomena such as rain, clouds, and fog, or scattering loss from the ground.

In the frame of a SAGIN communication link, the A2S narrow-band channel was theoretically investigated in [20], exploiting UAV as a relay with a LEO satellite. The stochastic model was based on regular geometry, in which the scatterers were distributed on a hemisphere and the VFM distribution was employed to jointly consider the azimuth and elevation angles, with the aim of resolving the statistical properties of the channel. These involved the space–time correlation function (STCF), the Doppler spectrum, the average fade duration (AFD) and the level crossing rate (LCR) of the channel. The results revealed that the moving direction of the UAV, the antenna direction, and the angle all significantly affect the statistical properties of the channel.

### 2.3. Rain Attenuation

**Figure 4:**
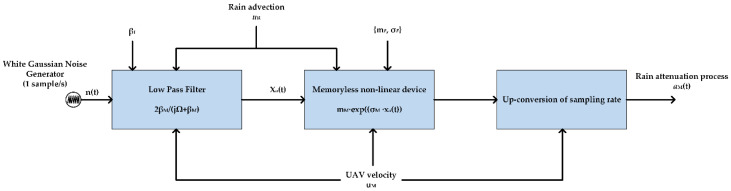
Layout of the rain-attenuation time-series synthesizer.

The A2S wireless link is expected to suffer from excess loss due to rain attenuation, especially if frequencies above 10 GHz are adopted for their operation, according to ITU [7]. Therefore, it is pivotal to determine the rain attenuation for the A2S/S2A links at frequencies above 10 GHz, such as Ku and Ka bands, which are becoming increasingly attractive to researchers aiming to support mobile-broadband satellite services for UAVs. At these frequencies, rain is expected to compromise the channel viability. In this context, a stochastic channel model for predicting the dynamic properties of rain attenuation for A2S/S2A links is appropriately modified on the basis of [21,22]. There are many rain-attenuation synthesizers for fixed links, such as [23,24,25,26], based on different assumptions, but not for A2S and A2A links. At this point, a modified model is provided, in which particular emphasis is placed on their LOS-channel state. The fundamental assumption of the model provided is that the rain-attenuation process can be approximated by a first-order stochastic differential equation. The architecture of the rain-attenuation time-series synthesizer is presented in Figure 4.

**Figure 5:**
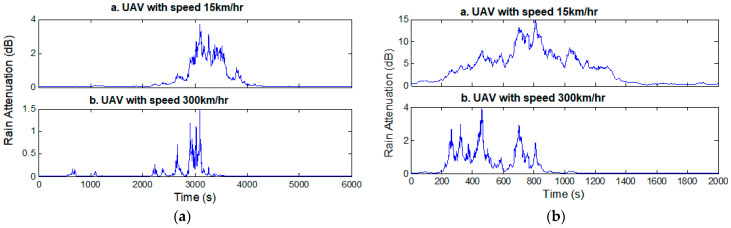
Simulated rain-attenuation time-series in Athens (GR), for UAVs at two different velocities compared with the fixed terminal: (**a**) Ku band; (**b**) Ka band.

The methodology described above is an extension of the Maseng–Bakken stochastic model of rain attenuation for fixed links [27], which was recently acknowledged as the most promising model for reproducing actual phenomena through time-series synthesizers. It is based on the basic theory of stochastic differential equations (SDEs). Next, the appropriate choice of parameters for the first-order proposed SDE model is investigated (model parameterization). This involves the calculation of static (i.e., long-term) and dynamic (i.e., short-term) parameters of rain attenuation. A key factor in the conversion of fixed rain-attenuation statistics into mobile rain-attenuation statistics is the velocity factor *ξ*, defined as [28,29]:

(4) $$\xi =\frac{u_{R}}{\left|u_{M}-u_{R}\cos\varphi \right|},$$

where *u_M_* is the amplitude of the velocity vector of the UAV terminal, *u_R_* is the amplitude of the velocity vector of the raincells (advection, or front speed), and *φ* is the relative angle between these two vectors. Both velocities are provided in km/h. Figure 5 presents simulated rain-attenuation time-series in the Ku and Ka bands, produced for UAVs traveling at speeds that range from pedestrian (15 km/h) to high (300 km/h).

As one can observe in Figure 5, only frequency affects the rain, with higher losses yielded in the Ka band (up to 15 dB) than in the Ku band (up to 4 dB) for the same UAV speed (15 km/h). On the other hand, higher attenuation values are induced at lower speeds, yielding, for example, up to 4 dB for 15 km/h and up to 1.5 dB for 300 km/h, respectively, in the Ku band. This is attributable to the fact that the UAV is moving faster than the raincell.

**Figure 6:**
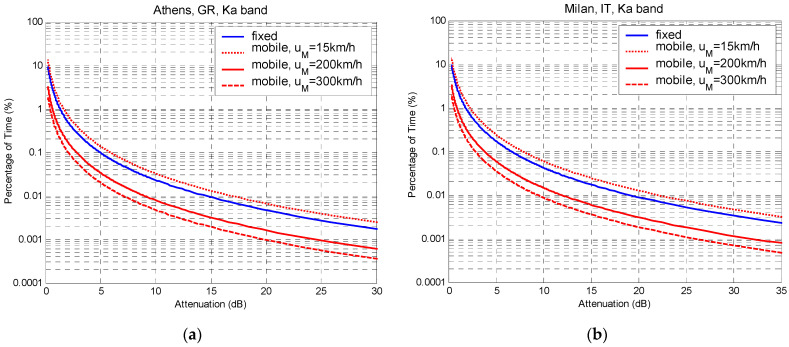
Long-term exceedance-probability distribution of rain attenuation for UAV terminals: (**a**) Athens, GR, in Ka band; (**b**) Milan, IT, int Ka band.

Consider, as an example, the geostationary satellite, HELLAS-SAT (39° E), which provides coverage to UAVs in two different European cities (Athens, GR, and Milan, IT). To simplify the analysis, let the UAV be in rectilinear motion. In addition, the worst-case assumption for the angle *φ* between the front-speed vector of the raincell movement and the velocity of the UAV is adopted, that is, when both travel in the same direction (*φ* = 0°). The implementation of the model for the Athens and Milan cases in the Ka band is illustrated in Figure 6. The average front speed of the movement of the raincells for Athens is 51.3 km/h, and for Milan, it is 30 km/h. The distribution of a fixed terminal is compared with those of a UAV terminal, moving at three different velocities (15 km/h, 200 km/h, and 300 km/h), ranging from very low to very high. For a given outage probability, as the UAV velocity increases, the required fade margin decreases. However, if a UAV preserves a velocity lower than the advection of the raincells, the required fade margin increases compared to the fixed-satellite system.

The time-series synthesizer in Figure 4 can also be applied in A2A wireless links. In this case, the *u_M_* in (4) takes into account the combined vector from the velocities of two UAVs. Therefore, $u_{M}=\sqrt{u_{a}^{2}+u_{b}^{2}-2u_{a}u_{b}\cos\theta }$, where *u_a_* and *u_b_* are the velocities of the first and second UAV, respectively, and *θ* the relative angle between their velocity vectors. If one of the UAVs is stationary, then *u_M_* = *u_a|b_*. The results for the attenuation of the A2A channel are similar to those presented above, since the raincell advection is now associated with the combined velocity vector. Since the proposed model is a physical mathematical model, its limitations lie in its basic assumptions, which are mostly related to the statistical distribution of the rain attenuation on a fixed link, which is considered lognormal distribution. Obviously, this needs experimental validation. With the employment of new UAS systems and the launching of satellites with new technologies, experimental campaigns on the evaluation of the rain attenuation in these links are expected.

### 2.4. Additional Theoretical A2S Studies

For the sake of completeness, additional theoretical studies described recent advances that do not elaborate on channel models. The optimization of the UAV trajectory and deployment was addressed in [30,31], aiming at achieving efficient transmission efficiency in the A2S link. Furthermore, adaptive channel-tracking algorithms and beam-tracking for the A2S channel were investigated in [32,33,34]. Finally, the link-level performance regarding the packet backlog, delay, and throughput as a function of the UAV number and link utilization was examined theoretically in [35], in scenarios in which UAVs accessed satellites.

Finally, Table 1 summarizes all the related research regarding the A2S channel, including experimental, theoretical, and simulation studies.

## 3. Air-to-Air Channel Models

The A2A communication scenario features cases in which more than two UAVs establish links within a group. These multiple drones communicate with the ground stations (one or more) through a backhaul channel with either one or multiple UAVs. The A2A channel has not been extensively studied in terms of experimental measurements [1], especially considering large UAVs. The channel resembles free space, with a dominant LOS component and regular, low-power multipaths from ground reflections, yet it is directly reliant on the flight altitude and the surrounding area. Usually, the A2A channel exhibits flat fading characteristics at higher altitudes; however, depending on the UAVs’ relative velocities and the scattering environment, the channel can be extremely time-variant (i.e., inducing high Doppler spread), which entails a carrier-frequency offset and inter-carrier interference, which both severely affect the communication channel [36].

### 3.1. Experimental Campaigns

Wideband A2A channel measurements were carried out in [37], transmitting a 20-MHz signal at 250 MHz, with an output power of 10 W. Two rotor-propelled aircraft were employed for this purpose, carrying the Tx and the Rx. Three different flight altitudes were selected (600, 1600, and 2600 m), while the trajectories included different environment types, such as water, urban, forest, and grassland. It was found that the received-power-delay profile (PDP) comprised a direct LOS component, as well as a specular reflection from the ground, which was strongest when flying above a water terrain (lake). The measured delay spreads were found between 0.356 μs and 0.979 μs, depending on the terrain and the flight altitude. Furthermore, the observed Doppler spread was in the range of 10.66–23.23 Hz; the highest value occurred when the flight path was over the forest at low altitudes (600 m). In general, delays in the reflected component in the PDP and the Doppler spread conveyed the distance between the two aircraft. Finally, additional low-power components were observed in the Doppler profile at frequencies up to ±128 Hz. These peaks were independent of the terrain type and the flight altitude and were caused by reflections on the rotor blades, which were related to the number of rotor-blade revolutions. This indicates that apart from the UAVs’ relative motion, additional Doppler spread can be induced by the vehicles’ structures themselves, such as those of rotor-propulsion systems.

**Figure 7:**
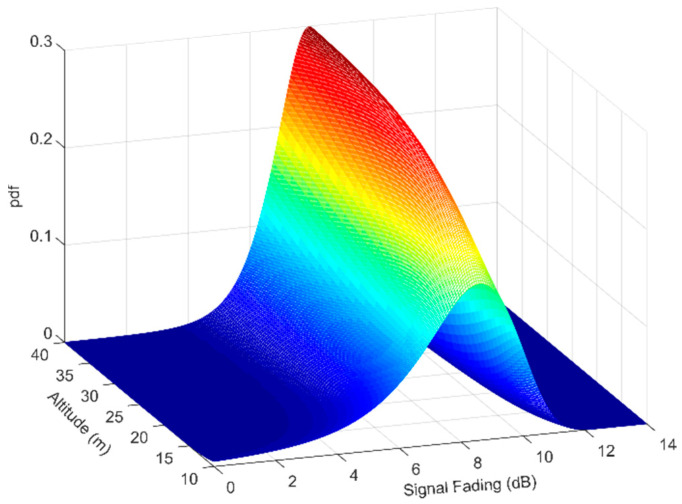
Probability density function of the Rician fading for various drone-flight altitudes.

Three different scenarios between two small UAVs (drones) for low altitudes (up to 50 m) were investigated for an IEEE 802.11n wireless link in [36]. The measurements were carried out at 2.4 GHz, with an output power of 20 dBm, and the received-signal strength was recorded at the Rx drone. The first scenario involved the two drones hovering at 35 m above the ground, and the fading properties were examined as a function of their separation. The results verified that the free-space-loss model accurately described the signal attenuation caused by the distance. The second scenario investigated the effect of the antenna directivity, in which the Rx drone hovered 25 m above the ground and rotated many times around the yaw axis. A significant variation in the received signal was observed, reaching up to 17 dB, which was associated to the drones’ antenna-radiation pattern. The final scenario was focused on examining the multipath effects in conjunction with the drones’ flight altitudes. The two drones were separated by about 50 m and hovered at 10, 25, and 40 m, respectively, above the ground. It was observed that at lower altitudes, there was a strong multipath from specular ground reflections, whereas the direct LOS component dominated at higher elevations. In this context, the authors proposed an extension of the Rician channel model in which the strength of the multipath power *σ* was modeled exponentially according to the following expression:

(5) $$\sigma =\alpha h^{b}+c,$$

where *a*, *b*, and *c* are constant parameters obtained by fitting (5) to the measurements, and *h* denotes the flight altitude in meters. The constant values were *a* = 212.3, *b* = −2.221, and *c* = 1.289. Therefore, the K-factor (direct to multipath ratio) of the Rice distribution can be described by

(6) $$K=\frac{\rho^{2}}{2{\left(\alpha h^{b}+c\right)}^{2}},$$

where *ρ* is the signal strength of the direct component. The obtained K-factor values for different altitudes were between 3.5 and 10 dB, indicating that the LOS component becomes dominant and stronger at higher altitudes, whereas the multipath due to the ground reflection affects the channel less. Applying (6) in the probability-density function (pdf) of the Rician distribution, considering a signal fade between 0 and 14 dB, the distribution of the signal variation for different Rx altitudes between 10 and 40 m is depicted in Figure 7. It is evident that as the Rx elevates, the *σ* decreases exponentially, which entails a stronger LOS component (i.e., the pdf and the K-factor increase), implying that the multipath power from the ground reflections diminishes. Figure 7 also demonstrates that at higher flight altitudes, the effect of the ground terrain declines and the established wireless link preserves a stronger unobstructed direct component.

**Figure 8:**
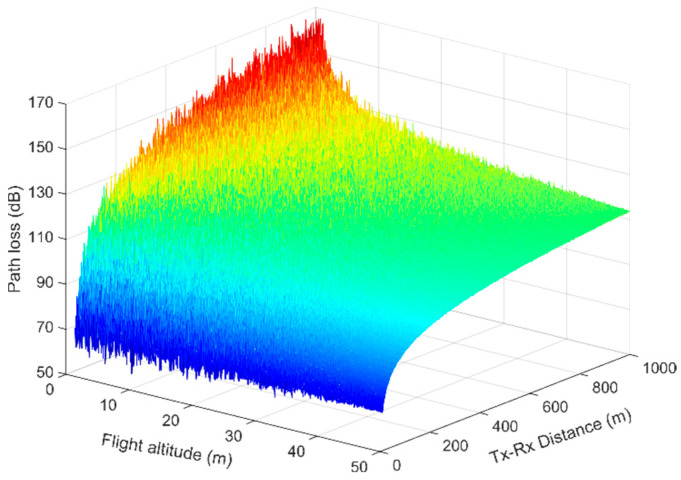
Path loss versus distance for different flight altitudes. The produced path-loss samples included the measured and the additional shadowing factor.

Path-loss measurements at 2.4 GHz between two small drones were carried out in [38]. Two different scenarios were examined. In the first case, the two drones were separated by about 10 m and then flew at a constant speed of 0.1 m/s, from the ground up to a height of 50 m. In the second scenario, the distance effect was investigated, in which the two drones hovered at 20 m above the ground, after which one remained steady and the other flew about 35 m away at a constant speed of 0.1 m/s. Based on the measurement results, a model similar to the close-in (CI) [9] was proposed (see also (13)), although the path-loss exponent was found to be associated with the flight altitude *h* according to *n* = *ah^b^* + *c*, where *a* = 2.6, *b* = −0.53, and *c* = 2, yielded by finding the best fit for the measured data. Moreover, apart from the shadowing factor, an additional fading parameter was recommended, also with a zero-mean Gaussian random variable. It was found to be related to the altitude, according to the linear relationship *X_h_* = *γh* + *δ*. The constant parameters were yielded from the best-fitting *γ* = −0.09 and *δ* = 4.7. The additional fading parameter implies that at higher altitudes, the standard deviation of the extra Gaussian distributed fading declines (in fact, it reduces linearly). A similar tendency can be observed in Figure 7, although in this specific scenario, the standard deviation reduced exponentially with the flight altitude. Therefore, the CI model can be rewritten as

(7) $$PL(d,h)=20\log_{10}⟮\frac{4\pi d_{0}}{\lambda }⟯+10n\log_{10}⟮\frac{d}{d_{0}}⟯+X(0,\sigma )+X_{h}(0,\gamma h+\delta ),$$

where, according to the measurement results, the standard shadowing factor *σ* = 1.9 dB. Applying (7) at 5.8 GHz, incorporating the additional fading parameter, the path-loss variation is illustrated in Figure 8. The drone separation is assumed to vary between 10 m and 1 km, with flight altitudes between 1 m and 50 m. In Figure 8, it is clear that as the flight altitude decreases, the shadowing factor becomes more intense, with a much greater magnitude. The ground-terrain effect accounts for this observation, as also explained previously, and increased fading is expected at lower altitudes. This tendency diminishes as the flight altitude increases, and at heights above 35–40 m, the fading magnitude is drastically limited (almost flat), as can be observed in Figure 8.

Furthermore, the path-loss exponent varies from 2.6 down to 2.3 for flight altitudes in the range of 1 m to 50 m. In other words, higher losses are expected at lower heights. This is more severe at altitudes below 15 m, where the path loss increases at a higher rate. For example, at a separation distance of 800 m, the path loss increases by almost 50 dB (from 100 dB up to 150 dB) when the flight altitude changes from 50 m down to 1 m. However, the path-loss variation relative to the drone height reduces down to 25 dB for smaller Tx–Rx separations (e.g., 200 m).

Additional experiments at millimeter-wave frequencies for the air-to-air channel were carried out in [39]. Two small hexacopters were employed to perform the measurements at a carrier frequency of 60.48 GHz, with a bandwidth of 2.16 GHz. The scenarios involved three different flight altitudes (6, 12, and 15 m above the ground) and 14 separation distances between the two UAVs, from 6 up to 40 m. The measuring equipment incorporated both Tx and Rx, arrays of 36 × 8 antenna elements, providing an angular coverage of 90° and 64 beam directions in the azimuth plane, which entailed a beam spacing of 1.4°. Two empirical models were proposed and compared: the CI model, which was previously described by (10), and the fixed intercept (FI) model, in which the path loss, in decibels, is expressed as

(8) $$PL=a+10\beta \log_{10}⟮\frac{d}{d_{0}}⟯+X_{\sigma },$$

where *a* is the floating intercept and *β* is the slope, which can both be yielded from fitting (8) to the measured data. The results showed that both models provide accurate prediction, with path-loss exponents equal to 2.25 and 2.33, and shadowing factors equal to 3.56 and 3.52 dB, for the CI and FI model, respectively. It should be pointed out that the FI model has no physical reference and simply fits to the measured data. Using the CI model, the impact of the flight altitude on the path loss was examined further. It was observed that both the path-loss exponent and the shadowing factor exhibited comparable values as the height increased. This indicated that the height did not influence the path loss during the UAV flight. Finally, the path-loss variation was examined by taking into consideration the beam misalignments. In this context, the FI model was fitted to the measured data for beam pairs that were not perfectly aligned. It was observed that the path loss increased progressively as the antenna misalignment between various beam pairs reduced the beamforming gain. For example, from perfect alignment between the best beam pairs (zero degrees) up to the highest misalignment of 4.2°, the floating path loss increased by almost 11 dB.

A large-scale statistical model for an A2A channel was proposed in [40], in which measurements were carried out at 5.8 GHz, employing two small-sized drones (hexacopters), which carried the equipment. The coupling effect of the antenna mounted on the drones was initially investigated by using an anechoic chamber. The antenna-radiation patterns were measured in the presence or absence of the air vehicles. The results showed that the coupling effect induced by the drones was insignificant and that the antenna patterns remained unaffected in both the azimuth and elevation planes. Furthermore, the measurements took place in a rural area with many trees in its terrain, inducing increased ground reflections. During the measurements, the Rx was stationary, whereas the Tx moved along a predefined route for distances between 25 and 425 m, and at a constant flight altitude of 50 m above the ground. The CI model, given in (10), was proposed to describe the large-scale characteristics of the A2A channel. The path-loss exponent was determined by establishing the time (obtained by averaging the power samples in the time domain) and frequency (obtained by summing the power samples in the frequency domain) and performing a time–frequency analysis (a short-duration Fourier transform was applied, after which an ensemble average was taken) of the measured data in terms of frequency-swept measurements using a 7-MHz bandwidth. The path-loss exponents were 1.817, 1.903, and 1.773, respectively, when applying each of these methods. Therefore, the final path-loss model is expressed as

(9) $$PL=\left\{\begin{array}{ll} 76.7-18.17\log_{10}\left(d/d_{0}\right)+13.26+X_{\sigma }, & \;\mathrm{Time}\\ 76.7-17.73\log_{10}\left(d/d_{0}\right)+0.5138+X_{\sigma }, & \;\mathrm{Time}-\mathrm{Frequency}\\ 76.7-19.03\log_{10}\left(d/d_{0}\right)-9.573+X_{\sigma }, & \;\mathrm{Frequency} \end{array}\right.,$$

where *d*_0_ is the reference distance selected (28 m) for the specific scenario (resulting in a free-space loss of 76.7 dB). The second constant values were the power offset relative to the free-space model. The shadowing factors were 0.52, 0.54, and 0.69 dB for the time, time-frequency, and frequency methods, respectively. The path-loss exponent was lower than that of the free space, which was attributed to the distinctive nature of the A2A channel and the drones’ characteristics. According to the results, the best fit is provided by the time-based method.

Finally, Table 2 summarizes all the related experimental campaigns regarding the A2A channel. For the sake of completeness, additional measurement campaigns are listed [41,42,43,44,45,46]; however, these are not analyzed further in the text.

### 3.2. Theoretical Studies and Simulations for Channel Modeling

Path-loss predictions for A2A links, exploiting the performance of machine-learning algorithms, were investigated in [47]. Simulations were carried out at 2.4 GHz, with Tx and Rx UAVs flying on predetermined routes over an urban environment. The Tx heights were set as 60, 70, and 80 m, whereas the Rx flew between 10 m and 40 m in steps of 10 m. Random forest (RF) and k-nearest neighbor (kNN) algorithms were employed to predict the path loss between the flying UAVs. When applying RF (using a tree-depth of three and one hundred and forty ensemble trees), the root-mean-square error (RMSE) between the simulated and the predicted path loss was 3.1 dB. On the other hand, the kNN (selecting k = 5 nearest neighbors) demonstrated a deteriorated RMSE, reaching up to 8.9 dB. However, both machine-learning methods outperformed the COST231 Walfish–Ikegami (28.5 dB) and Stanford University Interim (SUI), (13.4 dB) empirical models. Another significant finding was that the most important input feature that affected the predictability of the path loss was the path visibility (i.e., LOS or NLOS condition), with the minimum contribution observed for the Rx altitude.

The propagation characteristics of the A2A channel for urban and dense environments were studied in [48]. Full 3D ray-tracing simulations were conducted at 800 MHz and 2.4 GHz, in an area of 1.5 km × 1.5 km, transmitting a 100-MHz signal with a power of 0 dBm. The Tx UAV was set at 300 m above the ground and different Rx altitudes were studied in the range of 2–40 m. During the simulations, the direct, the reflected (up to 10 reflections), and the diffracted components were considered. The large-scale fading of the simulated channel was expressed in excess loss, given by

(10) $$PL_{\xi }(d,h_{\mathrm{Tx}},h_{\mathrm{Rx}})=20\log_{10}⟮\frac{4\pi d}{\lambda }⟯+n_{\xi },$$

where *d* is the Tx–Rx separation, *h*_Tx_ and *h*_Rx_ denote the flight altitudes of the Tx and Rx UAVs, respectively, and *λ* stands for the transmitted wavelength. In the following, the term *ξ* indicates whether there is LOS or NLOS in the propagation channel. The excess loss is normally distributed *n_ξ_*~*N*(*μ_ξ_*,*σ_ξ_*), with a mean value *μ_ξ_* and a standard deviation *σ_ξ_*, which are both were expressed as a function of the elevation angle and the UAVs’ altitude for either the LOS or the NLOS condition. The elevation angle between the Tx and the Rx is *θ* = sin^−1^((*h*_Tx_ − *h*_Rx_)/*d*). It was found that *μ_ξ_* increases exponentially with the Rx altitude, according to

(11) $$\mu_{\xi }=a_{\xi }\exp(b_{\xi }h_{\mathrm{Rx}}),$$

which stands for both the LOS and the NLOS condition, whereas the constant parameters were found to fit the data. On the other hand, the standard deviation was provided by

(12) $$\begin{array}{l} \sigma_{\mathrm{LOS}}=a_{\mathrm{LOS}}\theta +b_{\mathrm{LOS}}\\ \sigma_{\mathrm{NLOS}}=a_{\mathrm{NLOS}}{\left(\theta -b_{\mathrm{LOS}}\right)}^{2}+c_{\mathrm{NLOS}} \end{array},$$

where *a*, *b*, and *c* were yielded from fitting the simulated data and can be found in [48]. Equation (12) indicates that the standard deviation varied in a manner that was inversely proportional to the elevation angle. At low elevation angles, the wireless link between the UAVs was more likely to encounter shadow fading from the surrounding buildings, thus increasing the *σ_ξ_*. Furthermore, the CI model was found to approximate the simulated path loss well.

(13) $$PL_{\xi }(d,h_{\mathrm{Tx}},h_{\mathrm{Rx}})=20\log_{10}⟮\frac{4\pi d_{0}}{\lambda }⟯+10n_{\xi }\log_{10}⟮\frac{d}{d_{0}}⟯+X_{\xi },$$

where *d*_0_ denotes the reference distance (usually set as 1 m) and *n_ξ_* indicates the path-loss exponent. The latter was found to be related to the Rx height, with the same relationship as (11). Finally, *X_ξ_*, stands for the shadow fading, which is lognormally distributed with zero mean and standard deviation *σ_ξ_*. Similarly, the standard deviation depended on the elevation angle, according to (12). The path-loss exponents and the shadow fading were 1.98 and 2.54, respectively, and 0.9 and 9.43 dB for the LOS and NLOS conditions, respectively.

**Figure 9:**
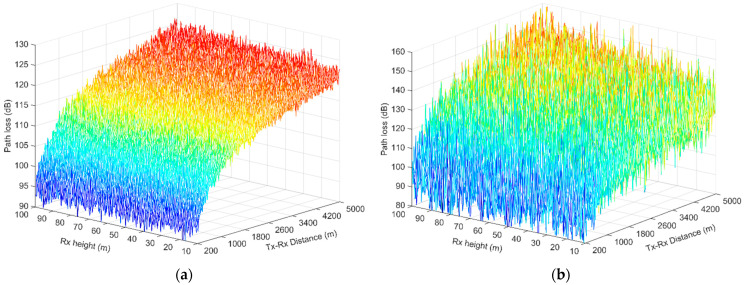
Path loss versus distance for various t Rx drone heights: (**a**) LOS condition; (**b**) NLOS condition. The produced path-loss samples included the shadowing factor.

Applying (11) and (12) in (13) for a dense urban environment, the path-loss variation for Tx–Rx distances up to 5 km is illustrated in Figure 8 for the LOS and NLOS scenarios, respectively. The Rx height was set between 10 m and 100 m. Higher losses were encountered in the NLOS condition and the shadow fading presented greater dynamic range, as can be observed in Figure 9b. At a distance of 5 km, the link in the NLOS conditions demonstrated a path loss between 130 dB and 150 dB (depending on the Rx elevation), which was 10–25 dB higher than in the LOS case. The flight altitude influenced the path loss less in the LOS condition, in which the Rx was close to the Tx and became more intense at greater Tx–Rx separations (the path loss was almost stable with the Rx flight at short distances, but varied linearly in the range of 5 dB at distances greater than 2 km). Similar tendencies were observed in the NLOS condition, although at greater distances, the path loss was affected more by the Rx height, demonstrating a linear variation in the order of 20 dB at distances greater than 2 km.

**Figure 10:**
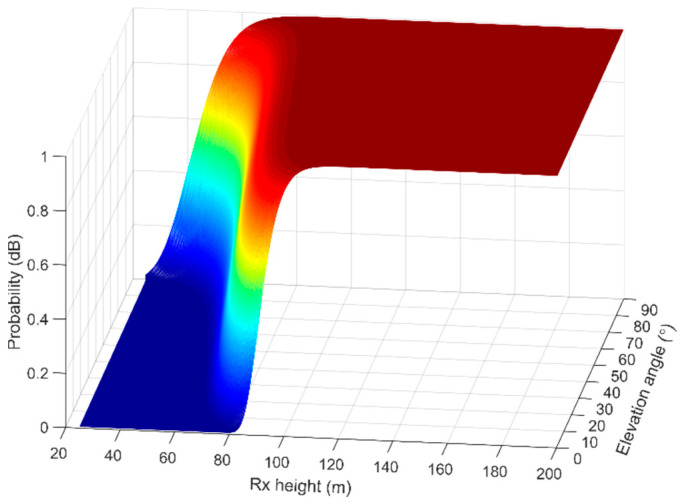
Indicative paradigm of LOS probability in Athens city center, when varying the elevation angle and the Rx flight altitude.

Another interesting approach is the probability of the LOS component between the two UAVs, which is directly associated with the altitude of the A2A link. Considering a geometrical deployment of buildings, the LOS probability is approximated by

(14) $$p_{\mathrm{LOS}}(h_{\mathrm{Rx}},\theta )\approx \exp⟮-\kappa Q⟮\frac{h_{\mathrm{Rx}}}{\gamma }⟯\cot(\theta )⟯,$$

where *θ* indicates the elevation angle in degrees between the two UAVs, *h*_Rx_ stands for the height of the Rx, and *Q*(∙) designates the *Q*-function. The parameter $\kappa =4\gamma \sqrt{2\alpha \beta /\pi }$ denotes the decay factor, which is correlated with the empirical parameters in the ITU-R model [49], which suggests that the probability of a LOS component depends on the building distribution. In the *k* parameter, *α* indicates the ratio of the land area covered with buildings to the total land area (dimensionless), *β* denotes the mean number of buildings per unit area (buildings/km^2^), and *γ* is a variable that regulates the building-height distribution. Taking into consideration Athens’ city center (dense urban environment), selecting *a* = 0.5, *β* = 300, and *γ* = 20 [49], and applying (14), the LOS probability for the specific terrain is indicatively presented in Figure 10. The elevation angle is set in the range of 0°–90° and the Rx flight altitude is set between 20 and 200 m.

Figure 10 indicates that the probability of achieving LOS between the two UAVs increases exponentially with the elevation angle, although it depends on the Rx height relative to the buildings’ height distribution. The highest probability is achieved for Rx heights above 80 m, which is about four times the height distribution of the buildings in the area under study (20–21 m, in Athens), and it is also independent of the elevation angle. To achieve, for example, an 80% probability for LOS links, an elevation angle close to 90° is required for flight altitudes below 60 m. However, to sustain a similar probability for lower elevation angles, the UAV has to fly higher, approaching altitudes of 80 m and above. According to Figure 10, it can be inferred that a safe approach to achieving a robust link between two UAVs in an urban environment is to maintain a flight altitude much higher than the average height of the buildings in the area. This can also be applied, as a simple rule of thumb, in other terrain types (e.g., suburban and rural areas).

A simple A2A path-loss model that takes into account the mobility of UAVs is presented in [50] for rugged terrains. It is based on the Stanford University Interim (SUI) model [51,52], extending its usage by incorporating additional losses due to the induced Doppler shift. The path loss, in decibels, is expressed as

(15) $$PL=20\log_{10}⟮\frac{4\pi d_{0}}{\lambda }⟯+10⟮a-bh_{\mathrm{Tx}}+\frac{c}{h_{\mathrm{Tx}}}⟯\log_{10}⟮\frac{d}{d_{0}}⟯+\Delta L_{f}+\Delta L_{h}+s+PL_{\mathrm{Doppler}},$$

where *λ* is the transmitted wavelength, in meters, *d*_0_ stands for the reference distance, *h*_Tx_ designates the Tx height, and *d* is the Tx–Rx distance, all of which are provided in meters. Furthermore, Δ*L_f_* = 6log10(*f*/2000) denotes the frequency-correction factor, in which the frequency, *f*, is given in MHz, *s* indicates the shadowing parameter, in decibels, due to NLOS conditions, and Δ*L_h_* is the Rx correction height, which is given by

(16) $$\Delta L_{h}=\left\{\begin{array}{ll} -10.8\log_{10}(h_{\mathrm{Rx}}/2), & \;\mathrm{Terrain}\;\mathrm{Type}\;A,\;B\\ -20\log_{10}(h_{\mathrm{Rx}}/2), & \;\mathrm{Terrain}\;\mathrm{Type}\;C \end{array}\right.,$$

where *h*_Rx_ is the Rx height, in meters. The coefficients *a*, *b*, and *c*, are terrain-specific and can be obtained experimentally [51]. The shadowing parameter is optional and is usually left to users (between 8.2 dB and 10.6 dB). Finally, the excess loss, in decibels, due to the Doppler is provided by

(17) $$PL_{\mathrm{Doppler}}=-3.72+5\log_{10}(f_{d})+5.01\log_{10}(d),$$

where *f_d_* is the Doppler shift (*f_d_* = *u*/*λ*) in Hz, and *u* is the velocity of the Rx towards Tx, in m/s.

**Figure 11:**
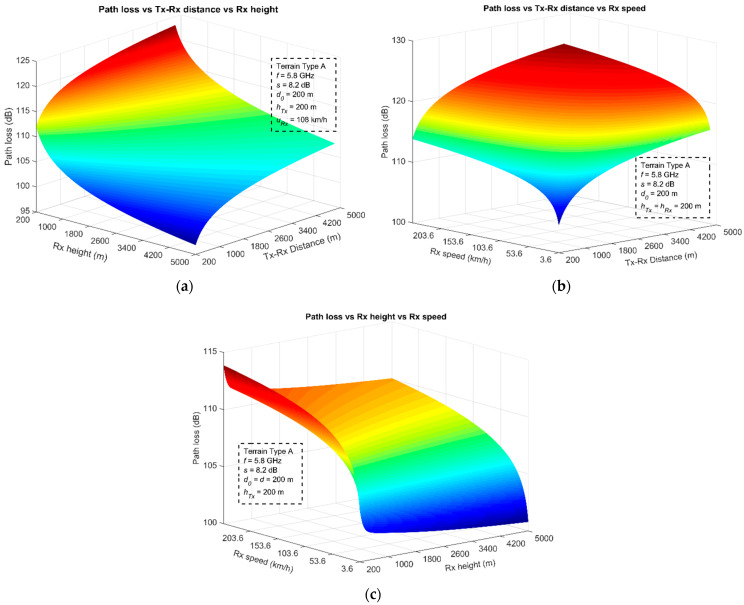
Path-loss effects with different parameters: (**a**) Tx–Rx distance and Rx height; (**b**) Tx–Rx distance and Rx speed; (**c**) Rx height and speed.

The following scenarios are presented in the form of a case study, in order to examine the impact of different parameters on the path loss. Applying (15) for a type-A terrain at 5.8 GHz and *s* = 8.2 dB, the variation in the path loss is examined in Figure 10 as a function of the UAV separation, Rx height, and speed. Figure 11a presents the path-loss variations for the UAV-separation distance, and Rx flight altitudes between 200 m and 5 km. The Rx is assumed to fly at a constant speed of 108 km/h, whereas the Tx hovers steadily at 200 m above the ground. The plot indicates that the path loss is influenced more by the Rx height because, at higher altitudes, it reduces drastically. This is attributed to the Rx correction factor Δ*L_h_*, which has a negative impact by decreasing the path loss as the Rx flies at higher altitudes. For example, even at the greatest separation between the two UAVs, of 5 km, the path loss is reduced by about 20 dB as the Rx height increases from 200 m up to 5 km. This observation is directly associated with the ground-terrain effects (e.g., reflection, diffraction, and scattering), as also mentioned previously, and at lower altitudes, the communication channel has a lower probability of establishing a LOS link and the signal-fading variations are also increased due to multipath, which entails increased attenuation. Furthermore, Figure 11b demonstrates the Doppler effect on the path loss, based on the speed of the Rx. In this specific scenario, both UAVs are positioned 200 m above the ground, whereas the Rx moves away from the Tx at various speeds, between 1 m/s and 70 m/s (i.e., from 3.6 km/h up to 252 km/h). The induced Doppler effect is more than evident, since, at higher speeds, the path loss escalates significantly. The specific variation can reach up to 10 dB and is virtually constant for distances of 200 m or 5 km between the two UAVs. The wireless-link range is influenced the most by the movement. For example, considering a viable link established at 4 km for a low-speed UAV (3.6 km/h), if the airborne vehicle accelerates up to 200 km/h, then the corresponding range should be reduced by about 91.2% (down to 351 m) in order to achieve convenient communication (i.e., to encounter the same path loss).

Finally, Figure 11c demonstrates how both the Rx speed and the height influence the path loss. The Tx is assumed to hover steadily at 200 m above the ground. The path-loss variation can reach up to 13 dB with the adjustment of these two parameters. However, the Rx speed is the most critical factor. As the UAV accelerates, the path loss escalates rapidly compared with its flying height. At low flying altitudes, the ground effect is still present, providing higher attenuation values. The corresponding path-loss variations are in the order of 2.5–3 dB, which is much lower than those induced by the speed of the UAV, indicating that the Rx speed is more dominant.

Another analytical A2A channel model that takes into consideration both LOS and NLOS conditions for dense urban environments was proposed in [53]. The specific model was developed using the elevation angle and the height difference between the transmitting and receiving UAVs. It is based on the two-ray-reflection model, as well as on knife-edge diffraction. The corresponding path loss is calculated, in decibels, according to

(18) $$PL=20\log_{10}(h_{\mathrm{Tx}}-h_{\mathrm{Rx}})+20\log_{10}(f)-20\log_{10}(\sin\theta )-147.55+L_{\mathrm{excess}},$$

where *f* is the frequency in Hz, *θ* corresponds to the elevation angle, and *h*_Tx_ and *h*_Rx_ denote the flight altitudes of the Tx and Rx UAV, respectively. The additional term, *L*_excess_, denotes the extra losses induced by the terrain, in either LOS or NLOS conditions. The excess loss is given by

(19) $$L_{\mathrm{excess}}=20\log_{10}\left(10^{\left(L_{\mathrm{LOS}}/20\right)}p_{\mathrm{LOS}}+10^{\left(L_{\mathrm{NLOS}}/20\right)}(1-p_{\mathrm{LOS}})\right),$$

where *L*_LOS_ and *L*_NLOS_ denote the extra losses, in decibels, if the LOS or NLOS condition is encountered, respectively, and *p*_LOS_ is the LOS probability provided by (14). The extra loss in the LOS condition can be calculated based on the two-ray model and can be expressed by the following relationship:

(20) $$L_{\mathrm{LOS}}=-20\log⟮2\sin⟮\frac{2\pi }{\lambda }\frac{h_{\mathrm{Tx}}h_{\mathrm{Rx}}}{h_{\mathrm{Tx}}-h_{\mathrm{Rx}}}\sin(\theta )⟯⟯,$$

where *h*_Tx_ and *h*_Rx_ denote the flight altitude of the Tx and Rx UAV, respectively, *θ* stands for the elevation angle, and *λ* is the transmitting wavelength. The excess loss for the NLOS condition can be found by using the Ikegami knife-edge diffraction model [54], and it can be calculated as follows:

(21) $$L_{\mathrm{NLOS}}=10\log_{10}(f)+10\log_{10}(h_{\mathrm{Tx}}-h_{\mathrm{Rx}})+20\log_{10}(\cos(\theta ))-10\log_{10}⟮1+\frac{\sqrt{2}}{L_{r}^{2}}⟯-68.8,$$

where *L_r_* is the reflection loss, and the rest of the parameters were described previously. The reflection loss takes values between 4 dB and 15 dB, on average [54].

**Figure 12:**
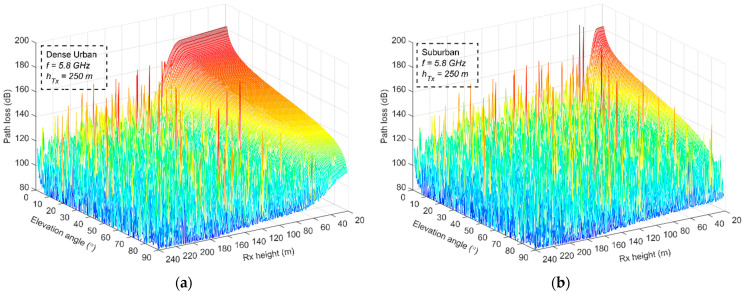
Indicative path-loss results when varying the Rx flight altitude and the elevation angle for two different propagation scenarios: (**a**) dense urban environment; (**b**) suburban environment.

The aforementioned model is applicable if the flight altitude of one of the UAVs is higher than the average building height in the area (i.e., *h*_Tx_ > *γ*). Let *h*_Tx_ = 250 m, *f* = 5.8 GHz, and *L_r_* = 6 dB; the path loss is calculated for variable *h*_Rx_ distance and elevation angles, applying (18)–(21). Two different propagation scenarios are considered. A dense urban environment in Athens’ city center, and Marousi, a suburban area a few kilometers north of Athens. The parameters for the LOS probability are selected according to [49], with *a* = 0.5, *β* = 300 and *γ* = 20 for the first scenario and *a* = 0.1, *β* = 750 and *γ* = 8 for the second. The corresponding results are presented in Figure 12.

The flat area in the mesh plots corresponds to the NLOS condition, whereas the remaining area with the rapidly varying path loss and peaks is attributable to the behavior of the two-ray model in the LOS condition. The effect of the environment is more than evident, since in suburban locations, the probability of the NLOS condition is limited, as can be observed in Figure 12b. The Rx flight altitude is the primary parameter that controls the path loss. In urban locations, NLOS conditions are encountered for low flying altitudes (below 40 m) as the elevation angle approaches 90°, but as *h*_Rx_ increases, the transition from NLOS to LOS can be achieved at lower elevation angles and at distances greater than 40 m and up to 100 m. These are the Rx height limits after which the LOS condition is dominant. On the other hand, in suburban areas, these heights are much lower and LOS conditions can be achieved for Rx heights above 20–40 m, almost independent from the elevation angle. The path-loss behavior in both plots is directly associated with the mixed excess loss given by (19) and the LOS probability provided by (14). It is worth mentioning that LOS conditions are exhibited for Rx flight altitudes between 2 and 5 times, and between 2.5 and 5 times, the areas’ average building height, in urban and suburban locations, respectively.

In addition to measurements, stochastic models for the A2A channel have also been developed. For this purpose, geometry-based stochastic models (GBSMs) have been employed to investigate the non-stationary characteristics of the wideband A2A wireless link. A three-dimensional (3D)-cylinder geometrical model that combined a LOS path with single- and double-reflected components was recommended in [55,56]. The specific model involved two moving UAVs in the 3D space, with two antenna elements 0.5λ apart in both the Tx and the Rx. The simulations were conducted at 3 GHz, considering a cylinder radius of 300 m, a separation distance of 150 m between the UAVs, and a Tx height of 150 m. Two different scenarios were examined, yielding the time-frequency correlation of the channel (from the time-varying transfer functions). In the first, a constant speed (10 m/s) and trajectory were considered (linear motion), whereas, in the second, a variable speed and trajectory were involved (applying a Gauss–Markov model for the randomness). In both cases, the Tx and Rx moved vertically and horizontally in the 3D space.

In the first scenario, based on the time-correlation function, it was observed that when the UAV moved vertically, the correlation time decreased faster compared with the horizontal movement (between 1 ms and 2.5 ms, depending on the selected reflections). This was true for both single- and double-bounce cases, implying that the vertical movement affected the channel stationarity more significantly. In this case, non-stationary characteristics were induced in the channel much earlier. In the case of double-bouncing reflections, the time correlation decreased faster in both the vertical and the horizontal movements of the UAVs. These observations indicated that, generally, the direction of movement affected the channel stationarity less in the double-bounced reflections; however, it demonstrated a direct influence when only single-bounce reflections were considered. In these cases, the wide-sense stationarity (WSS) of the channel cannot be easily preserved. Examining the time dispersion of the channel in terms of the frequency-correlation function, it was observed that no impact was induced by either movement direction (vertical or horizontal). On the other hand, the Doppler spectrum exhibited variations, which were affected by the UAV direction. When a UAV moved vertically, a sharper Doppler spectrum was created, whereas the Doppler shape widened if the horizontal movement was selected.

In the second scenario, in which arbitrary movement was applied, different characteristics were observed in the time-correlation functions. These exhibited significant changes in both time and delay. The channel stationarity varied between 1 ms and 4 ms, indicating that when the random motion increased, the channel became non-stationary much more rapidly. Comparing the movement directions, both exhibited similar time correlations in the early period, but after a certain time period, the horizontal movement resulted in a correlation with faster time, and after a few more seconds, the vertical movement exhibited fast non-stationarity. This clearly indicated that the channel stationarity is different in variable time instants. Furthermore, the frequency-correlation function still preserved similar characteristics in the second scenario, demonstrating that the arbitrary UAV speed compromises WSS only in the time domain. Finally, assessing the Doppler spectrum in different time instants of the UAVs mobility, it was found that in the early period, the spectrum demonstrated similar characteristics in both the vertical and the horizontal direction of movement. However, at later time points, the form of the spectrum changed significantly (becoming wider), again suggesting that arbitrary mobility reduces the stationarity of the A2A channel. In general, movement directions do not have a considerable impact on the channel correlation or the Doppler spectrum when significant random mobility is introduced to the wireless link.

The non-stationary characteristics of an A2A channel exploiting a GBSM model were also investigated in [57]. The specific model involved two UAVs that moved arbitrarily in a 3D space, incorporating two antenna elements in both the Tx and the Rx. The geometrical model comprised a LOS component, as well as scattering paths that arrived in clusters. These clusters underwent to an evolution process. Therefore, the non-stationarity of the channel was characterized by a birth–death process in those clusters, and a discrete Markov process was exploited for this purpose. The simulation was carried out at 2, 2.5, and 5.8 GHz, with an initial UAV separation of about 1 km. The velocity was set at 30 m/s for both the Tx and Rx, whereas 50 rays were assumed in each cluster. With an initial number of 10 clusters, it was observed that the random movement of both UAVs resulted in a fast cluster transition, the majority of which exhibited limited active duration. It was found that both the temporal and the spatial correlation functions decreased (from 20 ms down to 8 ms, and from 11 cm down to 4 cm, for 0.5 correlation levels) when the carrier frequency increased. The UAV antenna arrays accounted for this observation, since higher correlations were achieved when using lower carrier frequencies. Furthermore, the terminals’ motion pattern was found to substantially affect the channel stationarity. When the UAVs were close to each other, the time correlation was smoother, resulting in a higher correlation and stationarity time (of more than 25 ms for a correlation level of 0.5), which could reduce the channel estimation costs. On the other hand, the channel exhibited quick non-stationary characteristics (lower than 2.5 ms) if the UAVs travelled in different directions. Finally, the stationarity interval was calculated for various Tx speeds in the range of 15–45 m/s. The results revealed that the channel’s stationarity interval was drastically limited when the velocity escalated (from 0.4 s down to 0.05 s for a correlation level of 0.5), which was expected, because higher speeds entail rapid channel variation.

Finally, Table 3 summarizes all the theoretical studies and simulations regarding the A2A channel.

## 4. Challenges and Future Research Areas

### 4.1. Airframe Shadowing and UAV Machinery

Airframe shadowing and the ambient conditions of UAVs themselves are still unexplored and very intriguing research fields. As explained in Section 2, airframe shadowing can severely compromise the LOS link, inducing total connection and synchronization loss. In particular, for A2A links in which the elevation angles between the UAVs are in the order of 0–3°, different maneuvers of the wings and tail (e.g., U-turns or large pitch-and-roll turns) can block the LOS path. For example, signal-shadowing events up to 12.5 dB were observed in 45° rolls, in which the wings blocked the direct component. Diffraction and shadowing events were also found in a wavy UAV movement, which resulted in additional fades up to 2–3 dB. It was also shown that the antenna directivity may also be affected, for example, in yaw turns, in which substantial signal variations up to 17 dB were observed. These were associated with changes in the UAV-antenna-radiation pattern. Therefore, additional tests are required with small and large UAVs in order to measure and model possible airframe-shadowing phenomena related to the kinetic patterns of UAVs.

Furthermore, it is worth investigating the ambient conditions, which include vibrations from the UAV motor, air friction, turbulence, and sudden air gusts during flight. These specific phenomena may cause limited shadowing events and partial link losses (e.g., due to antenna misalignment), or a total link loss if the LOS path is blocked between the terminals’ antennas. Apart from the terminals’ speeds, Doppler effects are also induced by the reflections from the rotor blades, which are associated with the number of rotor-blade evolutions. These effects may increase the Doppler spread further and cause synchronization problems. In this context, additional studies and measurements are necessary not only for UAVs with rotor-propulsion systems (with single or multi-rotor engines), but also for large jet-propulsion vehicles. The latter may generate significant vibrations and jitter that can appear as additional peaks in the Doppler spectrum, thus increasing the spread.

Doppler spread can originate carrier-frequency offset and inter-carrier interference, particularly if orthogonal frequency division multiplexing (OFDM) schemes are adopted. Frequency synchronization can be exploited to combat these Doppler events and specific channel-access algorithms (e.g., multi-carrier code-division multiple access) can be employed to sufficiently alleviate Doppler spread. Nevertheless, further assessments are required through the performance of studies and measurements investigating these effects, which are introduced either by the UAV itself (as described above) or through different speed and motion trajectories. Of course, different and higher frequencies have to be investigated, because the existing results are related to low frequencies, thus emphasizing Ka and Ku bands, where Doppler effects are expected to be more intense.

Engines and ambient phenomena can also induce jittering, which directly affects the delay spread of the channel (the channel becomes more frequency-selective). Therefore, it is necessary to deepen knowledge on this topic by taking into consideration the physical structure of the air vehicle, its stability during flight (flight dynamics), the impact of the engine (single- or multi-rotor, jet), the position and type of the airborne antenna, and additional electronic payloads. Extensive measurements and thorough theoretical studies are then pivotal in order to evaluate those challenging effects, especially for unmanned UAVs at low and higher altitudes.

### 4.2. Antenna Diversity

Antenna diversity is another research field that requires further study. The application of spatial diversity at ground stations is not sufficient to counterbalance the potential shadowing events when UAVs maneuvers. The placement of multiple antennas and their orientation onboard a UAV is one solution to this problem. However, it is challenging to optimally place these antennas in order to minimize the shadowing effects, as well as the air-frame and motor noise during the flight. This is quite cumbersome and probably vehicle-specific. Moreover, customized antennas should be selected and studied in terms of their mechanical feasibility onboard UAVs. These should be compact, lightweight, low-profile, and able to preserve specific aerodynamic standards (e.g., fin-shark-type and patch antennas are more appropriate for mounting on wingtips). The number of mounted antennas necessary to optimize the antenna diversity depends on the operating frequency (e.g., millimeter waves are more appealing for compact antennas), the UAV volume, and the mission environment. Multiple antennas may be favorable, as they enhance coverage and provide sufficient diversity gain; however, there is a significant need for greater computation, space, and power supply.

### 4.3. Applicability of MIMO Antennas

The applicability of multiple-input–multiple-output (MIMO) antennas that provide sufficient multiplexing gain and spectral efficiency in A2A and A2S links is limited, for many reasons. The lack of a scattering environment in the proximity of the airborne terminals is one reason why these antennas are only capable of offering minimal throughput, which makes them comparable with single-input–single-output (SISO) architectures. In addition, it is rather problematic to mount multiple antennas onboard small UAVs with sufficient element separation to improve the multiplexing gain. On the other hand, the exploitation of millimeter-wave carriers could be a solution to accommodate small-sized antenna arrays, albeit with the drawback of increased path loss. Furthermore, MIMO antennas require high power consumption, which is another limitation, especially for small-sized battery-powered UAVs, as well as the fact that the MIMO multiplexing gain can be further degraded because it is cumbersome to obtain precise channel-state information in time-variant airborne links, especially when terminals fly at lower altitudes.

Both A2A and A2S links require clear LOS conditions to establish viable communication. In this regard, the application of beamforming techniques is very promising, especially when millimeter-wave frequencies are utilized. Therefore, convenient beam alignment would enable the Tx and Rx to select beams that maintain the maximum beamforming gain, thereby alleviating severe path loss and improving the channels’ link budget. However, rapid and precise beam tracking is very intriguing in airborne links with extreme mobility. Instead of beamforming, the establishment of free-space optical (FSO) links is an alternative solution, which could provide reliable communication in both A2A and A2S scenarios, although their deployment requires further study, making it a very significant theme for future research.

### 4.4. Interference Issues

Interference and channel allocation is another important issue that requires further study. Payload and CNPC are the two communication types that are used concurrently and incorporated in UAVs. However, they are not currently standardized, and both use distinctive operating bands, which may or may not coincide. The CNPC links are critical to ensure flight safety, and interference would be destructive. Moreover, they necessitate robust security measures and invulnerability to jamming or hacking transmissions. In any case, both the payload and the CNPC should operate in highly protected frequency bands, in order to ensure safe, secure, and interference-immune communications. Nevertheless, regulations and frequency assignments cannot always protect airborne links from interference, which would downgrade the communication efficiency of UAVs. Therefore, it is fundamental to evaluate and measure potential interference (usually unintentional), taking into consideration various environments. Interference measurements should be carried out in densely inhabited areas (e.g., urban and suburban environments), and in industrial environments, both of which present an increased likelihood of interference in different frequency bands. Different parameters have to be evaluated, such as the interferers’ location, the interfering power, the antenna diagrams, the duty cycle, and even various modulation schemes. All these provide valuable knowledge that will assist in implementing proper mitigation measures. Adaptive modulation and coding algorithms could be employed as measures against interference, especially when unlicensed bands are used. However, these do not perform appropriately in highly dynamic A2A and A2S channels, where mobility is introduced and stationarity conditions do not apply.

### 4.5. Channel Stationarity

As shown in Section 3 the mobility of UAVs significantly affects the channel stationarity, thus compromising the WSS characteristics. Non-stationarity is a fundamental characteristic that distinguishes UAV channels from ordinary channels. The inference of wide sense stationary uncorrelated scattering (WSSUS) is often disrupted in A2A and A2S communication. Apart from the geometrical stochastic models presented above, there is no extensive and thorough research available in the literature that assesses the non-stationary characteristics of the A2A and A2S channels. Moreover, the absence of measurements is more than evident. In this context, the assessment of the stationarity intervals will be an intriguing and novel topic in future research, in which the wideband-frequency-selective characteristics of the channels (small-scale fading parameters) will need to be evaluated within these stationary periods. Different metrics can be employed for this purpose, using various propagation scenarios, such as the time-domain power-delay profile (PDP) the correlation coefficient, the correlation matrix distance (CMD), the spectral difference, or spectrum evolutionary methods. Therefore, additional measurement experiments in different environments and different flight scenarios (with variations in altitude, speed, trajectory, and distance) will help to elaborate the stationary characteristics of the A2A and A2S channels further.

### 4.6. Measurement Campaigns

The presented case studies highlighted the need for further measurements for both A2A and A2S channels regarding various parameters that affect their communication performance. These include the carrier frequency, the underlying terrain, the flight altitude and dynamics, mobility scenarios, etc. Therefore, it is necessary to carry out measurements in new frequency bands, such as Ka and Ku bands, millimeter-wave, and sub-THz, which remain unexplored. In addition to built-up areas (dense urban, urban, and suburban environments), a variety of propagation terrains have to be taken into account, especially highly reflective surfaces, such as lakes and seas. These are highly likely to produce severe ground reflections, which entail increased multipath components and enhanced dispersive characteristics. Different velocities for one or more UAVs should also be tested, along with mobility randomness and trajectory scenarios. Current channel models rely on low flying altitudes; therefore, additional campaigns should be focused on unmanned vehicles at much higher altitudes. Excess loss can be induced by the atmosphere and rain, which can degrade the channel performance, especially if new proposed frequency segments are adopted (see examples above). Additional campaigns have to be undertaken in order to assess these effects. Despite the inherent difficulties, measurements during rain events would constitute optimal scenarios, which would provide significant information about the propagation characteristics in cases of precipitation, especially for long-duration UAV flights. Finally, in future 6G networks, THz and FSO frequency bands will be considered. This will be a novel and challenging field for future research, since channel measurements in these bands have still not been addressed.

## 5. Conclusions

This paper presented an extensive review of the available path-loss-prediction and channel models regarding the A2A and A2S propagation channels. State-of-the-art models were addressed and specific case studies were assessed in order to determine the inherent nature and behavior of those channels. According to the published results, both are highly influenced by many parameters, such as flight dynamics, distance and altitude, underlying terrain, velocity and trajectories, and, finally, atmospheric effects. A time-series rain-attenuation synthesizer that can be applied in both A2S and A2A links was presented, and the excess loss in the event of precipitation was evaluated. Finally, many intriguing research gaps were emphasized. These necessitate further measurements at new frequencies and flight altitudes, in propagation environments, with multielement antennas.

## Figures and Tables

**Table 1:** Relevant research on the A2S communication channel.

Refs.	Methodology	Frequency	Equipment	Delivered Research
[13]	Measurements	18.685 GHz	Airplane (Dornier 228 U-CALM) u = 250 kts	Airframe shadowing events
[14]	Measurements	L-band	Aviator 200 UAV u = 140 km/h	Throughput and latency tests
[15]	Measurements	1575.42 MHz	Aérospatiale Alouette III Sikorsky S-70 Black Hawk Lockheed C130 Hercules Pilatus Porter PC-6	Physical statistical model LOS + ground reflection
[16]	Theoretical	1.6 GHz	Aircraft u = 1600 kts	Channel-impulse response LOS + ground reflection + ground scattering
[17]	Simulations	THz	-	Path loss, including rain and cloud absorption
[20]	Simulations	n/a	UAV	Geometry-based model STCF, Doppler, AFD, LCR
[30,31]	Theoretical	2 GHz 2.4 GHz	UAV (u = 50 m/s) UAV (u = 0.1~40 m/s)	Trajectory optimization and UAV deployment
[32,33,34]	Simulations	Ka band	UAV	Beam tracking, 3D-channel tracking, adaptive channel-tracking algorithms
[35]	Simulations	n/a	Multiple UAVs	Link-level performance (packet backlog, delay, and throughput)

n/a: not available.

**Table 2:** Summary of previous experimental campaigns for the A2A channel.

Ref.	Signaling	Frequency	Equipment	Delivered Research
[37]	BW = 20 MHz P_Tx_ = 10 W	250 MHz	Aircraft Cessna C-208B Aircraft Dornier 228-101	Channel-impulse response, PDP, delay spread, Doppler
[36]	IEEE 802.11n P_Tx_ = 20 dBm	2.4 GHz	Small drones	Received-signal strength (RSS), antenna-directivity effects, Rice-model extension
[38]	IEEE 802.11n	2.4 GHz	Small drones (DJI Mavic 2 Zoom)	RSS, path loss, path-loss exponent, and shadow fading depend on flight altitude
[39]	IEEE 802.11ad BW = 2.16 GHz	60.48 GHz	Hexacopters (DJI M600)	Path-loss models (FI and CI), beam misalignments
[40]	BW = 7 MHz	5.8 GHz	Hexacopters DJI Matrice 600 Pro	Path loss based on time, frequency, and time–frequency analysis
[41]	BW = 20 MHz P_Tx_ = 10 W	250 MHz	Aircraft Cessna C-208B Aircraft Dornier 228-101	Delay and Doppler characteristics, surface-scatter characterization
[42]	IEEE 802.15.4	2.45 GHz	Hexacopters	Path-loss characterization (FI model with path-loss exponent 2.05), packet reception rate
[43]	IEEE 802.15.4 P_Tx_ = 60 mW	2.4 GHz	Fixed Wing (0.5 m wing span)	RSS, Path loss (FI model with path loss exponent 1.92), packet loss characterization
[44]	IEEE 802.15.4 P_Tx_ = 10 mW	2.4 GHz	Delta-wing UAV (0.8 m wing span)	RSS, path loss (FI model with path-loss exponent 0.93), packet error characterization
[45]	BW = 7 MHz P_Tx_ = 30 dBm	5110 MHz	Fixed Wing (2.8 m wing span)	RSS, PDP, time delay Model: LOS + multipath
[46]	BW = 20 MHz P_Tx_ = 20 dBm	2.375 GHz	Manned aircraft	Channel-impulse response, PDP, delay spread Two-ray model: direct + specular diffuse

P_Tx_: transmitted power.

**Table 3:** Relevant theoretical studies and simulations for the A2A communication channel.

Refs.	Methodology	Frequency	Signaling	Environment	Delivered Research
[47]	Simulations	2.4 GHz	BW = 100 MHz P_Tx_ = 15 dBm	Urban	Machine-learning path-loss prediction (RF, kNN algorithms)
[48]	Simulations	800 MHz 2.4 GHz	BW = 100 MHz P_Tx_ = 0 dBm	Urban/dense urban	Path-loss model (CI) LOS probability
[50]	Theoretical	2.4 GHz 5.8 GHz	-	Urban	Path-loss model (SUI extension) Excess loss due to Doppler
[53]	Theoretical Simulations	5.8 GHz	-	Dense urban	Path-loss model Two-ray + knife-edge diffraction
[55,56]	Stochastic (GBSM)	3 GHz	-	Cylinder model	Time–frequency correlation functions, Doppler spectrum, channel stationarity
[57]	Stochastic (GBSM)	2, 2.5, 5.8 GHz	-	Ellipsoid	Space–time correlation function, Doppler spectrum, channel stationarity

P_Tx_: transmitted power.

## Data Availability

Not applicable.
